# Care experiences of older people with mental health needs and their families in emergency medical services settings

**DOI:** 10.1111/opn.12500

**Published:** 2022-08-26

**Authors:** Deborah Goode, Assumpta Ryan, Vidar Melby, Paul Slater

**Affiliations:** ^1^ Ulster University Londonderry UK

**Keywords:** emergency Department, emergency medical services, experiences, families, mental health, older people, prehospital

## Abstract

**Background:**

There are challenges to person‐centred care provision in Emergency Medical Services (EMS) settings. The environment is often busy and noisy which can influence the experience of older people and their carer/partners when they attend emergency departments. Older people with mental health needs are a vulnerable group of people who are at risk of not having their needs met in acute care settings. This is due to complex presentations and increased pressures on the EMS system.

**Aim:**

The aim of the paper was to explore the care experience of older people with mental health needs and their carer/partners in pre‐hospital and in‐hospital Emergency Medical Services settings.

**Method:**

This study used an interpretive qualitative approach incorporating in‐depth, individual interviews to gather information on the experience of the older person with mental health needs and carers/partners. Data were analysed using Braun and Clarke's (2006) thematic analysis.

**Results:**

Fifteen individual interviews were carried out with older people with mental health needs (*n* = 10) and with carers/partners (*n* = 5). Six themes on ‘Getting there, getting in and getting out’, ‘Seeing the person’, ‘Perceptions and experiences of the pre‐hospital and Emergency Department (ED)’, ‘The effects of the experience on personal well‐being’, ‘Older person/carer/partner perceptions and experiences of the EMS staff’ and ‘Making it better’ emerged from the data.

**Conclusions:**

The results suggest that previous experiences with the emergency care system influence the way older people with mental health needs and their carers make decisions on current and future care needs. Negative experiences can be influenced by the layout and organisation of the ED. Participants remain reluctant to discuss or disclose their diagnosis in the Emergency Medical Services setting due to a perceived stigma. Health and social care systems and services need to undergo transformations to ensure that all people who access services are treated fairly and effectively.

**What does this research add to existing knowledge in gerontology?**

- There is a dearth of evidence reporting on the experience of older people with mental health needs and their carer/partners in Emergency Medical Services (EMS) settings. This study is the first of its kind to report the experiences of older people with mental health needs and their carer/partners in the pre‐hospital and in‐hospital emergency department settings.
- Older people with mental health needs report feelings of stigmatisation and these perceptions affect their well‐being and the judgements they make. Stigma influences their decisions to seek help and makes them hesitant to reveal their mental health needs to EMS staff. They delay seeking support for their physical and mental health needs, often making their condition/s worsen.

**What are the implications of this new knowledge for nursing care with older people?**

- We suggest that a priority triage category with a longer assessment time for older people with mental health needs is adopted. This would enable triage staff to assess and schedule treatment options, thus reducing waiting times. Older people with mental health needs may present with a complex history, comorbidity, and sensory or cognitive impairment. Increasing the time available to triage the older person would be beneficial for the staff to action a more detailed assessment.
- The creation of a standard assessment framework to assist in the assessment of mental health would assist in pre‐hospital and ED departments. Access to electronic care records, including the medical history and current medication, would assist triage nurses and paramedics in a triage decision for the attending complaint.
- There is an urgent need for training for the Multi‐Disciplinary Team (MDT) members on effective communication with older people who have mental health needs. Education should include the effect the physical environment (including sensory stimulation) can have on the older person with mental health needs (especially someone living with dementia). Education should include the family and their involvement in care and be co‐designed with older people/family members.

**How could the findings be used to influence policy or practice or research, or education?**

- The role of gerontological experts within EMS is vital in providing a person‐centred experience for older people (with and without mental health needs) and in education and development of the EMS staff to provide care. Emergency departments specialising in care of older people could help provide person‐centred care.
- Review of the built environment of EDs, such as access points and privacy considerations, to consider its impact on the older person with mental health needs. Support for the pre‐hospital staff and EMS staff in providing a new service delivery model should be a priority. This model could include the co‐created care pathways based on up‐to‐date evidence to offer the highest possible care for older people with mental health needs and their families.
- Education and training of EMS staff should be multidisciplinary, with a focus on both older people and mental health to ensure effective communication and continuity of care.
  Definition of terms
  Emergency medical services (EMS) refer to pre‐hospital emergency and emergency department care.
  Pre‐hospital care is any emergency response by the ambulance service to any setting.
  EMS staff include nurses, nursing auxiliaries, doctors, paramedics, ambulance technicians and other members of the multidisciplinary team.

## 1 INTRODUCTION

Emergency medical services (EMS) is the term used to refer to pre‐hospital emergency and emergency department (ED) care. Using these services can be a frightening and distressing experience for older people and their carers/partners as the environment is often noisy and busy. These issues can be exacerbated by longer waiting times in ED, as they often require more complex assessments and admission to hospital for further care (Goode et al., 2021). Vulnerable older people with mental health needs are at risk of not having their care needs met in acute care settings due to these complex presentations, ageism, lack of education and increased pressures within the EMS (Dewing & Dijk, 2016; Goldberg et al., 2012). The way the health and social care system has responded to rising numbers of attendances has presented a challenge to person‐centred care provision in ED (McConnell et al., 2016). An exploration of how the acute healthcare system meets the needs of older people with mental health problems, their carers/partners and the healthcare team is therefore beneficial in both education and healthcare planning.

### 1.1 Background

The World Health Organisation's definitions of the terms ‘older person’ and ‘mental health’ are used in this study. According to the WHO (2015), most Western societies consider the age of 65 and over to apply to an ‘older person’. Mental health is ‘… a state of well‐being in which an individual realises his or her own abilities, can cope with the normal stresses of life, can work productively and is able to make a contribution to his or her community’ (WHO, 2004, p38). The WHO (2017) report that worldwide, approximately 15% of people over sixty live with a mental health disorder. Consequently, as the numbers of people in society are getting older, the number of older people attending emergency departments across the world is increasing. The context of caring for older people with mental health needs in the pre‐hospital and acute care areas, such as ED, has been a source of question and concern worldwide. Traditionally, the focus in EMS is on the Triage process and treating life‐threatening physical presentations; consequently, mental health needs are not seen as high importance. A key factor to providing effective care in emergency medical settings is early assessment. To be able to holistically assess and treat this patient group within a brief period of time requires an elevated level of competence in both physical and mental health conditions (Mental Health Foundation, 2009). The primary focus of EMS staff is the culture of ‘fixing problems’ that are usually physically orientated and that mental health assessment and care are of a secondary priority. The RCEM (2021, p2) emphasise that:

*‘A patient presenting to ED with either a physical or mental health need should have access to ED staff that understand and can address their condition, and access to appropriate specialist services, regardless of their postcode, GP or time of arrival.’*

Despite the widely reported rising numbers of older people in society, this age group are not the most frequent users of EMS. According to NHS data (2020a), in the decade from 2010–2020, those aged 65 years and over have presented in smaller percentages (19.2%–22%) than the 15–34 (26.8%–30.1%) or 35–64 (30.4%–31.3%) age groups. However, these figures are not representative of outcomes, as older people account for between 51% (Hakenewerth et al., 2015) and 85% (Goode et al., 2021) of the admissions to acute areas, and between 20% and 72% of older people being transported by ambulance (Goode et al., 2021; Melby & Ryan, 2005). Older people with mental health needs have poorer outcomes of care when compared to the same age group who do not have any mental health issues (Mather et al., 2014; Sampson et al., 2009, 2013; Schnitker et al., 2011). Older adults, in general, spend a long time waiting in EDs (5 h) and those who also have a mental health issue wait 44 min longer (Goode et al., 2021). In other quality indicators, the care is unequal in terms of a longer length of stay, increased reattending, increased risk of falling, reduction in activities of daily living (ADL) and in the way they are perceived and treated by some healthcare staff. The rates of readmission and hospitalisation are also high which require appropriate care planning, discharge and continuity of care as they are more likely to be discharged to a long‐term care facility (Bradshaw et al., 2013). Increased functional decline in both physical and mental realms are evident as well as a marked increase in mortality, both whilst in hospital and following discharge (Adams et al., 2015). Despite the parity in the statistical estimates and projections, there has been little research and planning undertaken to establish the nature and extent of service that will be required in the future.

There is a dearth of evidence reporting on the experience of older people with mental health needs and their carer/partners in Emergency Medical Services (EMS) settings. Statistical estimates and projections have indicated an increased prevalence of mental health conditions and increased life expectancy (WHO, 2022). However, related research and planning into strategies for the most effective ways to develop health and social care services for older people is limited. This is necessary to proactively establish the nature and extent of EMS that will be required. An important part of this planning is to consider how the older person with mental health issues and their carer/partner experience EMS when attending for physical needs. The need to strategically plan for future person‐centred care delivery led to the research question; what are the experiences of older people with mental health needs and their carer/partners in EMS settings? This study was the first of its kind to examine this question. This paper reports on stage two of a larger study. Stage one data examined 74,766 ED attendance records of adults aged 65 years or over. Older people with mental health needs made up a sub‐sample of 1818. When compared to older people without mental health needs, the sub‐sample waited longer, were admitted to hospital in higher numbers and relied heavily on the ambulance service (Goode et al., 2021).

## 2 METHOD

This study used an interpretive qualitative approach incorporating in‐depth, individual interviews to gather information on why and how the experiences of the older person with mental health needs and carers/partners occurred. The person‐centred nursing framework (McCormack & McCance, 2010, 2017) was the underlying theoretical framework.

### 2.1 Participants and recruitment

The World Health Organisation (2015) definition of older people was used alongside the International Classification of Disease 10 (ICD‐ 10) (WHO, 1993) diagnostic codes to develop inclusion and exclusion criteria (Table 1).

**TABLE 1:** Inclusion and exclusion criteria for interview Older people and carer/partners

	Inclusion criteria	Exclusion criteria
Service users	English speaking males and females 65 years and over who: ‐have mental health needs (as assessed by the clinical team using a recognised mental health triage tool and clinical judgement). ‐have the capacity to give informed consent. ‐have used the Emergency Department of a hospital	Male and female 65 years of age or older who have long‐term health needs who also have an acute physical illness that requires admission to hospital for treatment. Male and female 65 years of age or older with acute mental health needs such as acute delirium or end‐stage dementia or any condition that would prevent them from being able to give informed consent (as assessed by the clinical team using appropriate mental health assessment tools).
Carer/ partner	English speaking. Male and female carers or partners of an older person (65 years or over) who has long‐term mental health needs and has used the Emergency Department of a hospital.	None

Recruitment was based in two major Emergency Departments within two Health and Social Care Trusts in a region of the United Kingdom (UK). The EDs were representative of both urban and rural populations. Recruitment took place through the Health and Social Care Trusts, charity, and voluntary sectors. Senior nursing staff in the Trusts and empowerment officers/managers in the charity and voluntary sectors functioned as gatekeepers and facilitated recruitment. All gatekeepers had preparation for their role, including the purpose of the study, written protocols and confirmation of ethical approval. If the potential participant met the inclusion criteria, they were informed by the gatekeepers of the purpose of the study and given an information letter asking for consent to be contacted by the researcher. Three days after attending the ED, the clinical team ensured the person was discharged home using the hospital database. Following confirmation, the researcher telephoned to ensure that they were willing to participate and arranged a suitable time and location for the interview to take place. Written informed consent was obtained by the researcher using participant information sheets (PIS) and consent forms. The researcher did not know of selection of potential participants until they gave permission to be contacted.

The aim of qualitative interviews was to gain insight into their individual experiences, and therefore, the numbers of participants became less relevant. Older people had a variety of mental health needs, including living with dementia, anxiety, depression and alcohol dependency. All the older people attended ED because of a physical illness (Tables 2 and 3). Data saturation was reached at a sample size of 15 service users (10 older people with mental health needs and 5 carers/ partners).

**TABLE 2:** Older person's mental health need/s and reason for attending ED

Older person pseudonym	Mental health need	Reason for attending ED
Helen	Enduring mental illness	Fall and head injury
Barbara	Living with dementia	Several attendances for cardiac condition, this attendance was for a fall with fractures.
Tom	Anxiety	Several attendances for abdominal pain
Megan	Living with dementia	Several attendances for falls
Roy	Living with dementia	Myocardial infarction
Rose	Depression Living with dementia	Fall and dislocation of shoulder
Judy	Depression Living with dementia	Stood on broken glass
Terry	Alcohol dependency Depression	Fall and head injury
Sam	Living with dementia	Fall
Eric	Depression Living with dementia	Eye injury

**TABLE 3:** Carer and their older person's mental health History and reason for attending ED

Carer/partner pseudonym	Older person's mental health need	Reason for attending ED
Kelly (carer)	Enduring mental illness with acute exacerbations and lives with dementia	Multiple admissions for falls and other conditions
Karen (carer)	Living with dementia	Several attendances for cardiac condition, this attendance was for a fall with fractures.
Joan (partner)	Anxiety	Several attendances for abdominal pain
Gloria (carer)	Living with dementia	Several attendances for falls
Joyce (carer)	Living with dementia	Abdominal pain

### 2.2 Data collection

One‐to‐one semi‐structured interviews were used to explore the experience of the EMS system from the service user and carer/partner perspective. The interviews ranged from 20 to 100 min in length. At participants' request, interviews took place in the older person's or carer's home as that was more comfortable for them. Design of the semi‐structured interviews was guided from the literature, the objectives of the study and questions that arose from stage one (Goode et al., 2021). The interview schedule was organised around the four key constructs of the person‐centred nursing framework (McCormack & McCance, 2010, 2017) (Table 4). The participants were asked open questions such as ‘How would you describe your experience of the environment of the ED for care of older people with mental health needs?’ Probing questions such as ‘can you tell me more about that?’ and ‘what did you mean by…?’ were used to elicit clear and in‐depth information. Service users highlighted their experiences from all EDs that they had attended.

**TABLE 4:** Interview Schedule for Older person and carer/family member

Step 1	Introduce self to participant and explain about nature of the interview. Inform participant that interview will be recorded for transcription and anonymity will be assured. Check informed consent has been signed and that they are happy to continue. Probing questions such as ‘can you tell me more about that?’ and ‘what did you mean by?’ will be used to elicit clear and in‐depth information. Ask participant to introduce self with first name for the tape.
Step 2	
Prerequisites/attributes 1. Was this your (or your relative's) first visit to an emergency department? Probe for detail. 2. What made you (or your relative) attend the emergency department? 3. How did you know something was wrong? 4. How did you decide which ED to attend? Probe for influencing factors. 5. How did you (or your relative) get to the emergency department? What factors influenced this decision? 6. Did you travel alone or accompanied? If accompanied by whom? 7. What happened when you arrived at the department? 8. Can you tell me about how you/your family member was assessed when you arrived in the emergency department? Did you inform the staff of MH history? Did they ask and record?	
Context and Delivery of Care (Environment and Care Processes) 9. Would you tell me about your experience of being in the pre‐hospital and emergency department? 10. What is it like to be a patient/carer or partner in the pre‐hospital and emergency department? 11. What aspect of care in the pre‐hospital and emergency department was most important to you? 12. How would you describe your experience of the environment of the pre‐hospital and ED for the delivery of your (or your relative's) care needs? 13. Did you inform the staff about your mental health history when they were doing their initial assessment; what was the response? Did they assess for your MH care needs? 14. How do you feel that the MH care needs were provided for in pre‐hospital and ED? 15. What would you say were/are your main concerns about being in ED? 16. Can you tell me about your experience of waiting in the ED? Probe for length of time, reason for waiting (referral, etc.). 17. To what extent, if at all, did your ED experience impact your mental health…probe for increased anxiety, etc 18. What happened after you (or your relative) were treated at the emergency department? (Admission/discharge).	
Results (person‐centred outcomes) 19. How did you feel about the staff who delivered the care to you or your partner? Probe for competency, caring, communication especially in relation to MH needs assessment (listening). 20. Did you feel like you (or your relative) had personalised treatment? 21. Were you involved in any of the decision‐making relating to your (or your relative's) care? 22. Did you feel satisfied with the care you (or your relative) received? 23. Is there any other comment you would like to make about your experiences or views on your experience of the pre‐hospital and emergency department?	
Step 3	Ensure participant has had opportunity to give opinions and thank them for their help in the study. Close interview.

### 2.3 Data analysis

Thematic analysis of the data followed Braun and Clarke's (2006) six phase method. This allows for identification, analysis, and reporting of the data in patterns and themes within a data set, with the researcher taking an active role in the identification/selection of patterns and themes and the reporting of the same. The interviews were recorded on a digital voice recorder and transcribed on a word‐for‐word basis. Following the transcription process, pseudonyms were used instead of real names to ensure the source of the data was unidentifiable to anyone other than the researcher. NVivo 10 (2014) data management system was used to organise and manage the data (Braun and Clarke Phase 1). During analysis, the researcher made notes in the transcriptions as both a memory aid and to assist in the coding process. Braun and Clarke's (2006) 15‐point checklist was completed within the research team and the six phases of thematic analysis achieved (Table 5).

**TABLE 5:** Ways in which Braun and Clarke (2006) 6 Phases of Thematic Analysis was achieved in this study

Step	Phase	Description of the process	Ways in which this was achieved in this study
1.	Familiarising yourself with your data	Transcribing data (if necessary), reading and rereading the data, noting down initial ideas.	All interview transcripts were read and reread whilst listening to the interview recording for correction to ensure accuracy of transcription.
2.	Generating initial codes	Coding interesting features of the data in a systematic fashion across the entire data set, collating data relevant to each code.	Paper copies of the transcriptions were viewed and initial codes highlighted using Saldaña's suggested questions on why they were chosen (2016)
3.	Searching for themes	Collating codes into potential themes, gathering all data relevant to each potential theme.	Interviews were uploaded to NVivo 10 (2014), and selected quotations coded into subthemes (Nodes)
4.	Reviewing themes	Checking in the themes work in relation to the coded extracts (Level 1) and the entire data set (Level 2), generating a thematic map of the analysis.	Word frequency and tag clouds generated in NVivo 10 (2014). Mind/code maps generated.
5.	Defining and naming themes	Ongoing analysis to refine the specifics of each theme, and the overall story the analysis tells; generating clear definitions and names for each theme.	Narrative exemplars for generation of each subtheme produced. Development of themes from subthemes and narrative exemplars. Consultation with supervisory team in peer review process
6.	Producing the report	The final opportunity for analysis. Selection of vivid, compelling extract examples, final analysis of selected extracts, relating back of the analysis to the research question and literature, producing a scholarly report of the analysis.	Review, development and redefining of themes

### 2.4 Ethics

Ethical approval was obtained from Ulster University and Office for Research Ethics Committees in Northern Ireland. This study adhered to the governance processes within the University and the Research Governance Framework for Health and Social Care and to the research management procedures of individual trusts and other public‐sector healthcare providers/organisations. Ethical principles as identified by International Council for Nurses (ICN, 2012) were adhered to during the study.

### 2.5 Ethical conduct

#### 2.5.1 Rigour and trustworthiness

Lincoln and Guba (1985) describe four areas that can assure that the findings of a qualitative study have rigour and trustworthiness as credibility, transferability, dependability and confirmability. How these areas were addressed in the study is shown in Table 6.

**TABLE 6:** Rigour and Trustworthiness in the study

Lincoln and Guba (1985) area	Ways in which this was achieved in this study
Credibility	Credibility was maintained through the use of using an ethically approved semi‐structured interview schedule to guide the discussion and the use of word for word transcriptions. A sample of these were also co‐analysed by a member of the research team. The principal investigator kept a research journal and memos on NVivo 10 (2014), with notes and reflections on issues as the study progressed.
Transferability	Transferability is demonstrated through the description of sampling factors including the geographical location of the study, the number of participants, the pseudonym of participants and their reason for attending ED (Tables 2 and 3), and the timeframe of data collection and analysis.
Dependability	Dependability is assured through the detailed information provided on the research method including inclusion and exclusion criteria (Table 1), participant information (Tables 2 and 3), interview schedule (Table 4) and process of thematic analysis (Table 5).
Confirmability	Confirmability of the results are demonstrated though peer review during thematic analysis and data reporting. Themes and discussions also include direct quotations from the participant and are confirmable through the process of coding into themes and the production of coding exemplars and theme maps (Lincoln & Guba, 1985). Braun and Clarke's (2006) phases of thematic analysis produced coding mind maps and narrative exemplars. Theme diagrams were created, and a joint review and analysis of themes was completed by the interview team.

## 3 RESULTS

Six themes emerged from the data. The voice of the participants shaped the themes and subthemes that are presented in Table 7. Each theme is reported using direct quotations from the participants.

**TABLE 7:** Themes developed from Older people with mental health needs and their carer or partner data

Theme	Subtheme
Getting there, getting in and getting out	Personal traits and attitudes of older people with mental health needs Decision‐making prior, during and after attending ED The time, culture and consequence of waiting
Seeing the person	Equality of treatment The importance of communication Stigma
Older person/carer/partner perceptions and experiences of the pre‐hospital and ED ‘Behind the magic doors’	ED system and organisation Layout of the department Privacy and confidentiality
The effects of the experience on personal well‐being	Emotional responses to the experience of being in ED Impact of ED on mental health
Older person/carer/partner perceptions and experiences of the EMS staff	Positive attributes of the EMS staff Lack of understanding and caring
Making it better	Need for physical and structural change User/carer involvement in planning Increase in numbers of staff and training System changes

Theme 1. Getting there, getting in and getting out.

Independence and remaining in control of their day‐to‐day lives was essential and decisions about how to get to the ED emphasised this point. Decisions related to how quickly they would be attended in their home and on arrival to ED. EMS staff were viewed as being able to sort out immediate problems, thus reducing the worry and the wait to be attended. In the ED, needs were met instantly whilst seeking help via the General Practitioner (GP) usually involved waiting for an appointment. There was a perception that ‘ambulance patients’ were prioritised over those who could walk into the department. This affected on decision‐making on what was the best way to attend the ED.

*‘…when we were in an ambulance…he was seen straight away…If you go in an ambulance, obviously you get into one of those rooms and you're seen straightaway…’* (Joan, Partner).

Many of the participants wanted to find out immediately what was wrong with them. However, once the older person was in the ED and they had some idea of how they were feeling and what was wrong, the priority changed to wanting to get out of the ED and go home.

‘…*let's go there, let's get the problem fixed and be discharged’* (Karen, Carer).

The time, culture and consequence of waiting were referred to as what Tom (older person) described as ‘*Waiting for God to see you’*. Time spent waiting in the ED is not a pleasant experience for anyone, but for older people who also have additional pressures because of their mental health needs, it became an unpleasant experience. The consequences of having to wait depended hugely on the reason they attended the ED, but also their existing mental health needs. For some participants, the waiting was frustrating but for others, it became impossible. Waiting in an environment that was noisy and crowded would make them unable to cope.

Theme 2. Seeing the person.

Older people wanted the EMS staff to consider them as individuals and not just focus on the mental health need. Communication was central to providing effective care in the pre‐hospital setting and ED. The older people viewed having mental health needs as one that still carried a stigma for them. Roy, who lives with dementia, explained how he felt the stigma of the diagnosis when he took physically unwell with a myocardial infarction and had to be sent to the ED in the ambulance:

‘*I didn't know who knew or who didn't know [about the diagnosis] at that time. And I was still feeling then was this stigma that's attached to this…do I or do I not tell them?’* (Roy, Older Person).

Stigma associated with a mental health diagnoses resulted in non‐disclosure of their mental health needs until they deemed it necessary for their care provision. Only two of the older people with mental health needs and one carer disclosed their diagnosis to the nurses in triage. Non‐disclosure was often because of the fear of the staff making a judgement about them.

Theme 3. Experiences of the pre‐hospital and ED.

The structure and organisation of EDs were confusing for some participants, and the constant movement of people around the department gave a sense of disorganisation. For some participants, the busyness related to the time of day and others believed staff were too busy to attend to them. One carer, Kelly, demonstrated her lack of understanding about what was going on when she described being called to be seen as going ‘behind the magic doors’. The experience of not knowing who your nurse or doctor was also confusing, with some participants describing their experience as traumatic, due to lack of knowledge from staff on how to support a patient with mental health needs.

Participants discussed treatment that they had received at one or more of the nine Emergency Departments throughout the region. The physical layout of the ED caused concern because of the lack of ability of the older people with mental health needs and their carer or partner to feel comfortable in that environment. Linked to the layout of the ED was privacy, which was reported by many of the participants. Hearing what was wrong with other people and them hearing what was happening with the older person was an issue for them.

*‘I think there's not enough privacy…for nurses coming out and saying something, you wouldn't want anybody else to hear…I heard every word that was going on…you don't want to hear people's business and people don't want to hear your business’*. (Judy, Older Person).

Theme 4. The effects of the experience on personal well‐being.

Emotions are often high for patients and family when there is an acute episode of illness, but when there was a lack of understanding or what appeared to be a lack of knowledge on mental health, it made the participants feel stressed, frustrated and angry. I explained issues around communication arose in situations where staff did not understand their mental health needs. The experience was frightening to some, often because of the fear of the unknown as Rose (Older Person who has depression and who lives with dementia) explained:

*‘It was nerve‐racking, I was lying on the bed like, but it was…it was nerve‐racking. I was worried because that's the first time I was ever in Casualty [ED]’*.

The noise and activity within the ED caused by other patients and staff caused tensions and fear. A feeling of panic and disorientation occurred due to the older person being transported about the department for tests, which was amplified by the numbers of people in the ED or by a feeling of being trapped. One carer also described the tension whilst waiting for something to be done but recognised the extreme feeling of relief once they took the older person in to see the doctor, knowing treatment would begin.

*‘But once she's in the bay and a doctor comes in, you nearly feel relief…it's somebody knows what they're doing’*. (Joyce, Carer).

One older person explained that any poor experience affected his confidence levels and that after this experience in the ED, the mental health after effects were worse than the physical illness he originally attended with. The primary focus appeared to be the physical needs, with less emphasis on understanding how the mental health needs were addressed. Roy, who lives with dementia, described how noise can have a stressful impact on his ability to concentrate on the discussion with EMS staff about his needs. The effect of the noise in the department frustrated him, and he became frustrated and angry.

*‘…There's no way in this wide world that I'm going through that again, I'd rather die. I'd rather die than go through that again…it had a serious mental effect on me personally…The physical part wasn't a problem, but the mental part… it's continuing to this day’*. (Roy, Older Person).

Noise is only one part of the sensory stimulation for the older person and carer, with the multi‐sensory stimulation of the whole ED having a detrimental effect on the mental health of the older person and carer/partner.

Theme 5. Older person/carer/partner perceptions and experiences of the EMS staff.

Views on interacting with the staff were polarised. Participants either thought the staff were amazing or terrible, angels or demons. Praise was extended to pre‐hospital staff who were viewed in incredibly positive terms, including an emphasis on how their presence whilst waiting to be dealt with in the ED made them feel safe. They were also viewed as providing significant support to the older people with mental health needs by being there for them, being friendly and helpful and providing a good standard of personalised care in a professional and knowledgeable manner. They could assess the problem and get it sorted, even though the ED was often busy. EMS staff tried to establish caring relationships showing understanding and compassion for the older person and family. (Karen, Carer) described good experiences with staff who were caring and compassionate.

*‘…you could see them trying to establish a rapport and they certainly displayed empathy and sympathy for her plight… They were very kind’*.

Some participants reported a negative experience of care from EMS staff, in which older people with mental health needs felt ignored.

*‘I think I could have died, and nobody would notice me sitting there. Not one person come in. Not one person to say, “Are you all right? Are you okay?”’* (Tom, Older Person).

At times, participants reported care from EMS staff as task orientated and lacking understanding of those with mental health needs. They felt they were ‘*just one of the crowd’* and ‘*boxes were being ticked’* as opposed to having individualised care.

Theme 6. Making it better.

Participants offered suggestions for physical and structural change in the department, with the proximity of other people and the lack of privacy as the key priorities. The waiting area caused most concern to many of the participants, and many participants suggested the provision of a quiet room that older people with mental health needs could use whilst waiting. Involvement of the users of the service was vital to making a strategic change when planning care structures for the older person.

The older people with mental health needs watched the care that was delivered in the ED, and they had a genuine understanding of the pressure the EMS staff were under. One older person put this into the context of how the lack of staff meant he felt very alone and could not speak to anyone of his concerns. They reiterated the need for training and education on how to recognise and care for older people with mental health needs several times throughout the interviews.

The system that allows unrestricted access to the ED is particularly important for the older people with mental health needs. However, the ‘availability’ of care 24/7 was mentioned as an area that concerned them because it added pressure to the ED system. Participants discussed the problems associated with waiting when the older person had mental health needs. All these participants had a physical need that was treated in the ED; however, one carer suggested that the system should be reviewed to allow the development of a special ED that catered for mental health needs including the need to streamline the bureaucracy and administration involved in the ED as it also contributes to the wait.

## 4 DISCUSSION

This study adds to existing evidence by reporting the emergency care experiences of service users and subsequent suggestions on how to improve EMS care. Two main overarching findings were elicited: firstly, the EMS environment and physical and mental well‐being and secondly, ongoing interdisciplinary education.

### 4.1 The ED environment

There is a need for change in the physical environment and a structural change in the system, including how people use EMS. Older people reported they want professional, competent care that includes information about what is happening and how long they will have to wait. Our respondents affirmed that attending the ED is a stressful experience that often causes fear and anxiety, which supports findings by Moss et al. *(* 2015) and McConnell et al. (2016). They have concerns about the design of the ED and its impact on care and their experience. The noisy, busy, crowded environment influenced their ability to cope with the situation, which supports findings from Watson et al. (1999), Bridges (2008) and the Royal College of Emergency Medicine (2018). Consistent with the findings of other studies (Dewing & Dijk, 2016) access, privacy, confidentiality and comfort in the ED were also discussed as major concerns. Waiting in the ED was always described as an unpleasant experience, with the noisy, crowded environment causing emotional and mental distress for both the older person with mental health needs and their carer/partner. Waiting appeared to emphasise a loss of control of the situation for the older people with mental health needs, who preferred to remain independent. All the participants knew that waiting was part of the healthcare system of emergency care. However, the very personal individual experience of waiting was apparent, which supports the findings from Considine et al. (2010). Consideration should be given to how long, why, and where the older people with mental health needs wait and that some planning is required to reduce the waiting time. The suggestions made by the participants echo the ethos of the specialised EDs with a focus on older people (Geriatric EDs) that have been established in the United States of America (USA) since 2008. Collaborative multidisciplinary guidance is provided on the use of equipment and supplies including appropriate layout, lighting and noise in ED (ECEP/AGS/ENA/SAEM 2013). If these areas were addressed they could contribute to the reduction of anxiety about confidentiality and crowding which were reported by the older people and their carer/partners. Guidance on the use of policies and procedures including triage, assessment and screening could ensure that the older person has the appropriate level of care to meet their needs. This would also have an impact on the waiting times that cause elevated levels of stress and anxiety in our participants. Similar older person's EDs (OPED) have been set up in the United Kingdom with Norfolk and Norwich University Hospitals NHS Foundation Trust being the first in 2016, and they have integrated many of these recommendations into their practice. This would be a welcome development.

### 4.2 Ongoing interdisciplinary education

Older people with mental health needs and their carers/partners wanted more ED staff and specific training for the EMS team. EMS staff require specific education and training to understand how to care for people who have mental health needs as well as they understand physical problems. It is recognised that the culture of ED is often ‘physical first’ and dealing with emergency lifesaving interventions. However, it is vital that strategic planning in EMS settings consider the relationship between physical and mental health. Education and awareness should include the information from the WHO (2017) Global strategy for mental health and the NICE (2016) guidelines on better access to ED for those with mental health needs. Supporting mental health in acute care areas, as mentioned in the care pathway, should be a priority, given the poor outcomes and inequality of care showed. This supports the recommendations from the RCP (2009) and NICE (2015, 2016) and previous studies by Goode et al. (2014) and Dewing and Dijk (2016). There have been educational developments in the form of a toolkit from The College of Emergency Medicine to improve mental health care (CEM, 2013) and competencies for older people and mental health care in the National Curriculum and Competency Framework for Emergency Nursing (RCN, 2017). The College of Paramedics also includes mental health in their latest curriculum guidance document (COP, 2017). It was also clear in the literature that more specialist knowledge and skills are required to care for older people (with and without mental health needs) in acute settings (Bunn et al., 2014; Faulkner & Law, 2015; Gray et al., 2013; Mental Health Foundation, 2009; Šteinmiller et al., 2015). The findings also support the need for specific training for all staff to understand the different care needs (including the environment) of people who are living with dementia and their carers, which was also clear in the review by Dewing and Dijk (2016). Collaborative multidisciplinary guidance in the United States includes recommendations on appropriate education and experience for staff to work specialised units for old age and the importance of continuing professional development (ECEP/AGS/ENA/SAEM 2013).

## 5 LIMITATIONS

The voice of older people with mental health needs have been missing in much of the literature, partly because it is a difficult population to recruit This was also the experience of the research team in this study who had to rely on gatekeepers to assist in recruitment.

The study was undertaken in one geographical region in the UK. Also, the number of participants was relatively small. Both of these factors may limit the transferability of the findings to other EMS settings.

### 5.1 Implications and recommendations

#### 5.1.1 United Nations sustainable development goals

Strategic preparation and readiness to respond to natural disasters is important given the impact such events can have on the use of EMS. Preparedness, risk assessment and continuity of emergency services should be planned, researched and developed to accelerate the appropriate response to ensure older people with mental health needs can continue to receive the care they need. Education and awareness of the culture of the ED environment of physical first should include the information from the WHO (2017) Global strategy for mental health and the NICE (2016) guidelines on better access to ED for those with mental health needs. Supporting mental health in acute care areas should be a priority, given the poor outcomes and inequality of care shown. This applies to the United Nations sustainable development goals (UN, 2015) number 3 (Good health and well‐being) and 10 (Reduced inequalities).

#### 5.1.2 Education

There is an urgent need for training for all MDT members on effective communication with the older person with mental health needs, including the effect the physical environment (including sensory stimulation) can have on the older person with mental health needs (especially someone living with dementia). This training should include the impact this has on the family and how they can be involved in care.

#### 5.1.3 Research

Further investigation into the causes of the inequality in outcome for the older person with mental health needs in acute care settings is recommended. Stigma would merit further exploration, examining why the older people with mental health needs still feel stigmatised. Examination is needed into the impact stigma has on their well‐being.

#### 5.1.4 Practice

Consideration should be given to the e impact of the built environment on the older person with mental health needs. This includes the access to the ED, including the ambulance, the physical environment and the sensory stimulation within the ED. The lack of privacy and confidentiality in the ED should also be considered. Older people with mental health needs and their carers/partners should be involved in this process.

Care pathways based on up‐to‐date evidence are needed to provide the highest possible care for older people with mental health needs and their families. Suggestions to include in the care pathway would include a combination of early senior doctor review, comprehensive assessment, a conducive and comfortable environment and early geriatrician and multidisciplinary team involvement (Wright et al., 2014). These should be monitored and developed alongside existing key performance indicators.

#### 5.1.5 Policy

The inequality of care that the older person with mental health needs experience should be recognised as a health and social care priority, with clear standards of improvement outlined for all health and social care providers. Co‐ordination of primary and secondary healthcare provision should consider the inequality of care and develop innovative ways of working to improve current provision to provide ‘joined‐up‐care’ for older people with mental health needs.

The development and implementation of national guidelines and staff education and training in the assessment and treatment of older people with mental health needs in EMS settings could ensure that the care experience is a positive one.

## CONFLICT OF INTEREST

The authors declare no conflicts of interest.

## Data Availability

The data that support the findings will be available in Ulster University PURE repository https://pure.ulster.ac.uk/ following an embargo from the date of publication to allow for full publication of research findings.
